# *Cornus mas* L. Extract Targets the Specific Molecules of the Th17/Treg Developmental Pathway in TNBS-Induced Experimental Colitis in Rats

**DOI:** 10.3390/molecules28073034

**Published:** 2023-03-29

**Authors:** Marta Szandruk-Bender, Beata Nowak, Anna Merwid-Ląd, Alicja Z. Kucharska, Małgorzata Krzystek-Korpacka, Iwona Bednarz-Misa, Benita Wiatrak, Adam Szeląg, Narcyz Piórecki, Tomasz Sozański

**Affiliations:** 1 Department of Pharmacology, Wroclaw Medical University, Mikulicza-Radeckiego 2, 50-345 Wrocław, Poland; 2 Department of Fruit, Vegetable and Plant Nutraceutical Technology, Wroclaw University of Environmental and Life Sciences, J. Chełmońskiego 37, 51-630 Wrocław, Poland; 3 Department of Medical Biochemistry, Wroclaw Medical University, Chałubińskiego 10, 50-368 Wrocław, Poland; 4 Bolestraszyce Arboretum and Institute of Physiography, Bolestraszyce 130, 37-700 Przemyśl, Poland; 5 Institute of Physical Culture Sciences, Medical College, University of Rzeszów, Towarnickiego 3, 35-959 Rzeszów, Poland

**Keywords:** experimental colitis, TNBS, Th17/Treg axis, IL-6, RORγt, STAT3, Foxp3, PIAS3, cornelian cherry, polyphenols

## Abstract

Given that one of the crucial events in the pathogenesis of inflammatory bowel disease is the loss of homeostasis between Th17 and Treg cells, targeting the specific molecules of the Th17/Treg axis developmental pathway is a promising strategy for inflammatory bowel disease prevention and treatment. The current study aimed to assess the impact of cornelian cherry (*Cornus mas* L.) extract, rich in iridoids and polyphenols known for their potential anti-inflammatory activity, at two doses (20 or 100 mg/kg) on the crucial factors for Th17/Treg cell differentiation in the course of experimental colitis and compare this action with that of sulfasalazine. This study was conducted on the biobank colon tissue samples collected during the previous original experiment, in which colitis in rats was induced by trinitrobenzenesulfonic acid (TNBS). The levels of IL-6, RORγt, total STAT3, *p*-STAT3, and Foxp3 were determined by ELISA. The expression of PIAS3 mRNA was quantified by qPCR. Cornelian cherry extract at a dose of 100 mg/kg counteracted the TNBS-induced elevation of IL-6, RORγt, and *p*-STAT3 levels and a decrease in Foxp3 level and PIAS3 mRNA expression, while given concomitantly with sulfasalazine was more effective than sulfasalazine alone in reversing the TNBS-induced changes in IL-6, RORγt, total STAT3, *p*-STAT3, Foxp3 levels, and PIAS3 mRNA expression. The beneficial effect of cornelian cherry extract on experimental colitis may be due to its immunomodulatory activity reflected by the influence on factors regulating the Th17/Treg axis.

## 1. Introduction

Crohn’s disease (CD) and ulcerative colitis (UC) are the two main entities of inflammatory bowel disease (IBD), with increasing incidence and prevalence not only in westernized countries but also in newly industrialized regions [1,2]. Both forms of IBD are chronic inflammatory disorders of the gastrointestinal tract, but whereas CD is characterized by transmural inflammation that may appear in any part of the gastrointestinal tract, UC is limited to inflammation of the colonic mucosa and submucosa [3,4]. IBD is characterized by alternating phases of clinical relapse with symptoms ranging from mild to severe and remission when symptoms may disappear [1]. Both CD and UC usually present with diarrhea, rectal bleeding, abdominal pain, low-grade fever, fatigue, unintended weight loss with reduced appetite, and anemia. As the disease progresses and the intestinal and extraintestinal complications develop, the differences between CD and UC patients become more and more evident [4]. Like many other chronic inflammatory diseases, CD and UC decrease patients’ quality of life, have a poor prognosis, and lead to lifelong morbidity [1,3,5].

Both CD and UC develop in genetically predisposed individuals as a result of an exaggerated mucosal immune response against intestinal antigens. Although the exact etiopathogenesis of IBD remains not completely understood, there is growing evidence that a shift in T helper 17 (Th17) and regulatory T (Treg) cells balance towards the Th17 cells contributes to the enhanced immune response in IBD patients [1,6]. Th17 cells are a proinflammatory subset that, in excess, promotes tissue inflammation. In contrast, Treg cells play a vital role in maintaining immune tolerance and suppressing excessive Th17 cell responses [7]. Both Th17 and Treg cells develop from naïve CD4+ T cells. Differentiation of Th17 cells from naïve CD4+ T cells is driven by their master transcription factor retinoic acid-related orphan receptor γt (RORγt) and signal transducer and activator of transcription 3 (STAT3) in the presence of proinflammatory cytokines, especially IL-6. IL-6 activates STAT3 by phosphorylation at tyrosine residue 705 [6,7]. Contrary, STAT3 transcriptional activity is negatively regulated by the protein inhibitor of activated STAT3 (PIAS3) by blocking its DNA-binding activity [8]. In turn, the differentiation of Treg cells requires the presence of forkhead box protein 3 (Foxp3) [6,7]. Dysregulation in these developmental pathways leads to an imbalance in the Th17/Treg axis and disturbance of immune homeostasis that underlie both experimental colitis and human IBD. Thus, targeting the developmental pathway of the Th17/Treg axis is one of the promising strategies for the pharmacotherapy of inflammatory bowel disease [9,10,11].

Recently, many plant extracts and their bioactive phytocompounds have been shown to regulate the Th17/Treg axis by targeting its developmental pathway molecules, primarily IL-6, RORγt, STAT3, and Foxp3. Many of these Th17/Treg axis regulators with great potential against IBD are polyphenols [9]. The cornelian cherry (*Cornus mas* L.) iridoid-polyphenolic extract (CE) seems interesting in this context. We have previously demonstrated that CE exerted a protective effect in experimental colitis in rats via attenuation of the intestinal inflammatory response [12]. Moreover, we have shown that CE decreased the level of IL-17, a crucial proinflammatory cytokine of the Th17 cell lineage, as well as the level of IL-23, a cytokine critically required for the maintenance, expansion, and pathogenicity of the Th17 cells [12]. With the above in mind, we decided to elucidate whether the beneficial effect of CE on the course of experimental colitis may be related to the Th17/Treg developmental pathway molecules.

The current study aimed to assess whether pretreatment with the cornelian cherry iridoid-polyphenolic extract influences the expression of the Th17/Treg developmental pathway-specific molecules as well as compare the action of CE with that of sulfasalazine, one of the standard drugs in IBD, and elucidate whether CE may act synergistically with sulfasalazine on the Th17/Treg developmental pathway-specific molecules.

## 2. Results

### 2.1. Chemical Characterization of Cornelian Cherry Iridoid-Polyphenolic Extract

**Figure 1:**
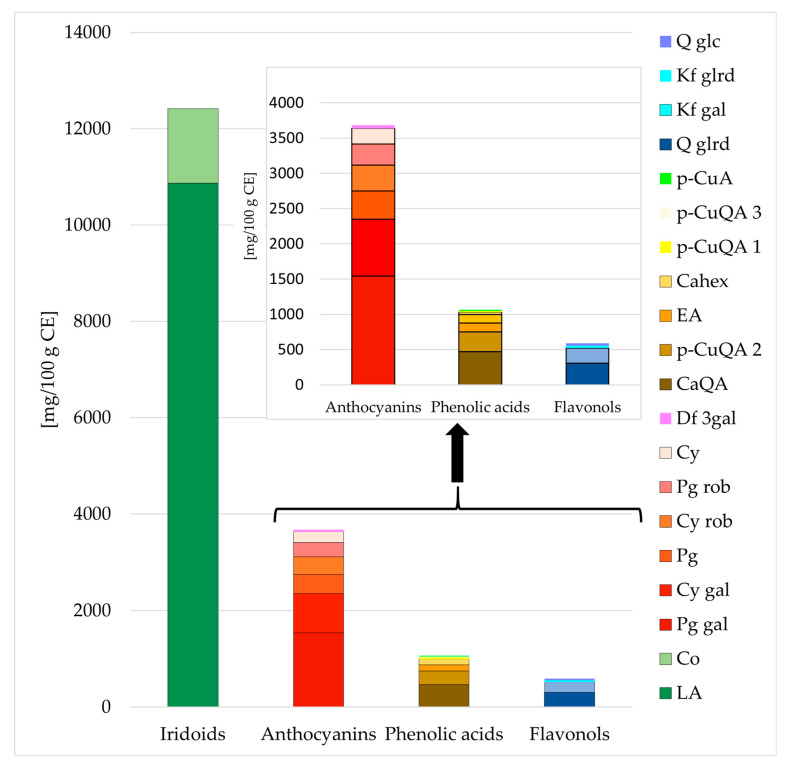
The content (mg/100 g dry mass) of the main compounds of the purified cornelian cherry iridoid-polyphenolic extract (CE) by HPLC. Abbreviations: LA–loganic acid, Co–cornuside; Pg gal–pelargonidin 3-*O*-galactoside; Cy gal–cyanidin 3-*O*-galactoside; Pg–pelargonidin; Cy rob–cyanidin 3-*O*-robinobioside; Pg rob–pelargonidin 3-*O*-robinobioside; Cy–cyanidin; Df gal–delphinidin 3-*O*-galactoside; CaQA– caffeoylquinic acid; *p*-CuQA 2–*p*-coumaroilquinic acid 2; EA–ellagic acid; Cahex–caffeoylhexoside; *p*-CuQA 1–*p*-coumaroilquinic acid 1; *p*-CuQA 3–*p*-coumaroilquinic acid 3; *p*-CuA–*p*-coumaric acid; Q glrd–quercetin 3-*O*-glucuronide; Kf gal–kaempferol 3-*O*-galactoside; Kf glrd–kaempferol 3-*O*-glucuronide; Q glc–quercetin 3-*O*-glucoside.

The predominant components of the studied extract, the detailed composition of which was demonstrated in our previous study [12], included iridoids, anthocyanins, phenolic acids, and flavonols (Figure 1). Using the HPLC method, 12,419 mg of iridoids, 3683 mg of anthocyanins, 1063 mg of phenolic acids, and 589 mg of flavonols in 100 g of CE were determined. Loganic acid, pelargonidin 3-*O*-galactoside, caffeoylquinic acid, and quercetin 3-*O*-glucuronide, respectively, prevailed in the above-mentioned groups of compounds, reaching 10,870 mg, 1542 mg, 471 mg, 306 mg, respectively, per 100 g of CE (Figure 1, Supplementary Materials: Figure S1).

### 2.2. The Effects of the Cornelian Cherry Iridoid-Polyphenolic Extract on the Concentration of IL-6, RORγt, Total STAT3, p-STAT3, and Foxp3 in the Colon Tissues

**Figure 2:**
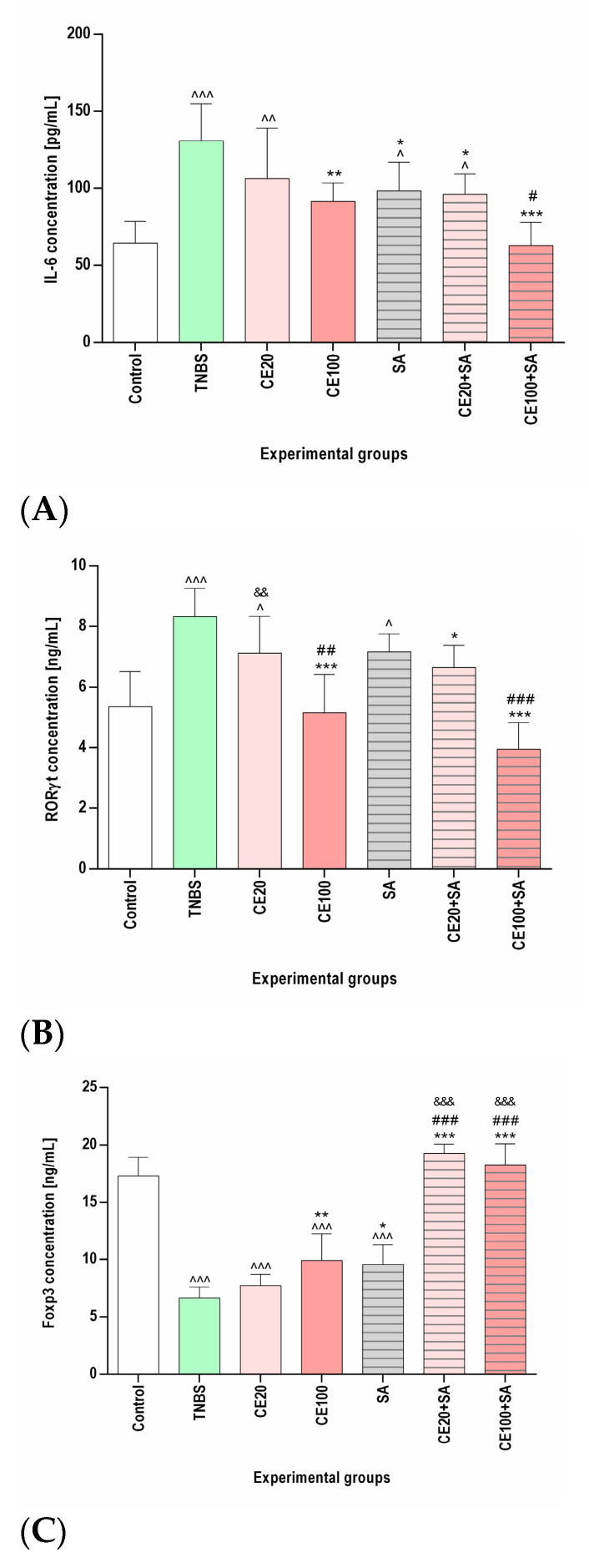
The impact of cornelian cherry iridoid-polyphenolic extract on the concentration of IL-6 (**A**), RORγt (**B**), and Foxp3 (**C**) concentration in the colon tissues in experimental groups. Control—control group; TNBS—group receiving only TNBS; CE20, CE100—groups receiving, respectively, 20 or 100 mg/kg cornelian cherry extract and TNBS; SA—group receiving 100 mg/kg sulfasalazine and TNBS; CE20+SA, CE100+SA—groups receiving, respectively, 20 or 100 mg/kg cornelian cherry extract and 100 mg/kg sulfasalazine and TNBS. Data are presented as mean values ± SD. Analyses were performed using a one-way ANOVA and Tukey’s *post hoc* test. Differences ^ *p* < 0.05, ^^ *p* < 0.01, ^^^ *p* < 0.001 vs. control group; * *p* < 0.05, ** *p* < 0.01, *** *p* < 0.001 vs. TNBS group; # *p* < 0.05, ## *p* < 0.01, ### *p* < 0.001 vs. sulfasalazine group; && *p* < 0.01, &&& *p* < 0.001 vs. CE100 group were deemed statistically significant.

As presented in Figure 2 and Table 1, TNBS increased the concentration of IL-6, RORγt, and both total and phosphorylated (activated) STAT3 (*p*-STAT3) in the colon tissues and decreased the Foxp3 concentration compared to the control group (*p* < 0.001, *p* < 0.001, *p* < 0.01, *p* < 0.001, and *p* < 0.001, respectively). Cornelian cherry extract at a lower dose (20 mg/kg) ameliorated the TNBS-induced changes in the concentration of IL-6, RORγt, total STAT3, *p*-STAT3, and Foxp3; however, the observed effect did not reach statistical significance (*p* = NS in all cases). At a higher dose (100 mg/kg), investigated cornelian cherry extract significantly reduced the TNBS-induced changes in the concentration of IL-6, RORγt, and *p*-STAT3 (*p* < 0.01, *p* < 0.001, and *p* < 0.001, respectively). In the CE100 group, normalization of the concentration of IL-6 and RORγt was observed, and the phosphorylation of STAT3 remained elevated compared to the control group (*p* < 0.01). No significant effect of cornelian cherry extract at a higher dose on the concentration of total STAT3 was observed (*p* = NS). Although cornelian cherry extract at a higher dose significantly increased the Foxp3 concentration (*p* < 0.01), it remained lower than in the control group (*p* < 0.001).

### 2.3. The Effects of Sulfasalazine Alone or in Combination with Cornelian Cherry Iridoid-Polyphenolic Extract on the Concentration of IL-6, RORγt, Total STAT3, p-STAT3, and Foxp3 in the Colon Tissues

Sulfasalazine significantly ameliorated the TBNS-induced elevation of the concentration of IL-6 and phosphorylation of STAT3 (*p* < 0.05 in both cases); however, they remained elevated compared to the control group (*p* < 0.05, *p* < 0.001, respectively). No significant effect of sulfasalazine on the concentration of total STAT3 and RORγt was detected (*p* = NS in both cases). In the SA group, similarly to the CE100 group, we observed the amelioration of TNBS-induced decrease in Foxp3 concentration (*p* < 0.05); however, it remained lower than in the control group (*p <* 0.001). The combined therapy with cornelian cherry extract at a lower dose and sulfasalazine, contrary to the monotherapy with the investigated extract at a lower dose, ameliorated the TNBS-induced elevation of IL-6, RORγt, and *p*-STAT3 concentration (*p <* 0.05, *p* < 0.05, and *p* < 0.01, respectively). In the CE20+SA group, we observed a normalization of the concentration of Foxp3 (*p* < 0.001), and the Foxp3 concentration was higher than in the CE100 (*p* < 0.001) and SA (*p <* 0.001) groups. The combined therapy with cornelian cherry extract at a higher dose and sulfasalazine seemed to be most beneficial, as in the CE100+SA group, the IL-6 and RORγt concentrations were normalized (*p* < 0.001 in both cases) and lower than in the SA group (*p* < 0.05 and *p* < 0.001, respectively). In the CE100+SA group, we observed a normalization of the concentration of both total and phosphorylated STAT3 (*p* < 0.001 in both cases), yielding a lower *p*-STAT3/STAT3 ratio than in the colitis group (*p* < 0.05). The effect of combined therapy with cornelian cherry extract at a higher dose and sulfasalazine on total and phosphorylated STAT3 was greater than that of sulfasalazine in monotherapy (*p* < 0.05 and *p* < 0.001, respectively). Additionally, in the CE100+SA group, Foxp3 concentration was normalized (*p* < 0.001) and significantly higher than in the CE100 (*p* < 0.001) and SA (*p* < 0.001) groups (Figure 2 and Table 1).

### 2.4. The Effects of the Cornelian Cherry Iridoid-Polyphenolic Extract on PIAS3 Expression in the Colon Tissues

**Figure 3:**
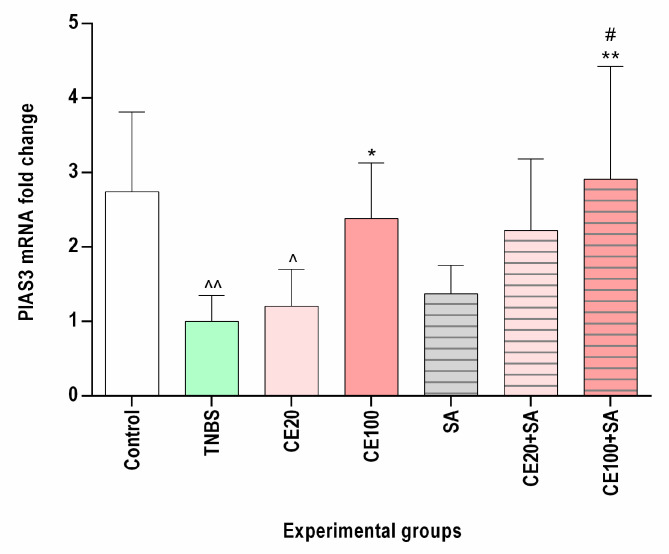
The impact of cornelian cherry iridoid-polyphenolic extract on the PIAS3 gene expression in the colon tissues in experimental groups. Control—control group; TNBS—group receiving only TNBS; CE20, CE100—groups receiving, respectively, 20 or 100 mg/kg cornelian cherry extract and TNBS; SA—group receiving 100 mg/kg sulfasalazine and TNBS; CE20+SA, CE100+SA—groups receiving, respectively, 20 or 100 mg/kg cornelian cherry extract and 100 mg/kg sulfasalazine and TNBS. Relative expression levels were analyzed by qPCR. Data are presented as mean values ± SD. Analyses were performed using the one-way ANOVA and Tukey’s *post hoc* test. Differences ^ *p* < 0.05, ^^ *p* < 0.01 vs. control group; * *p* < 0.05, ** *p* < 0.01 vs. TNBS group; # *p* *<* 0.05, vs. sulfasalazine group were deemed statistically significant.

As depicted in Figure 3, TNBS administration caused a decrease in intestinal PIAS3 mRNA expression as compared to the control group (*p* < 0.01). Cornelian cherry extract at a lower dose (20 mg/kg) resulted in a 1.2-fold increase in PIAS3 mRNA level as compared to the TNBS group, and this effect did not reach statistical significance (*p* = NS). Contrary, the studied extract at a higher dose (100 mg/kg) significantly elevated PIAS3 mRNA expression nearly 2.5-fold in comparison to the colitis group (*p* < 0.05). In the CE100 group, normalization of the PIAS3 mRNA level was observed.

### 2.5. The Effects of Sulfasalazine Alone or in Combination with Cornelian Cherry Iridoid-Polyphenolic Extract on PIAS3 Expression in the Colon Tissues

No significant effect of sulfasalazine on the PIAS3 mRNA expression was observed (*p* = NS). The combined therapy with cornelian cherry extract at a lower dose and sulfasalazine elevated the PIAS3 mRNA expression compared to the colitis group by 2.2-fold; however, the observed effect did not reach statistical significance (*p* = NS). In turn, the combined therapy with cornelian cherry extract at a higher dose and sulfasalazine resulted in a nearly 3-fold increase in the PIAS3 mRNA level in comparison to TNBS-subjected rats (*p* < 0.01). In the CE100+SA group, we observed normalization of PIAS3 mRNA expression, and the effect of such combined treatment was greater than the effect of SA alone (*p* < 0.05) (Figure 3).

## 3. Discussion

Despite the meaningful progress in therapy, IBD is still an incurable disorder, and the current frontline treatment has substantial limitations on safety and efficacy [13]. Therefore, there is still a need to develop new drugs with increased efficacy and reduced side effects, especially those based on pathogenic mechanisms targeted molecules that confer susceptibility to intestinal inflammation, with a particular focus on those that present therapeutic opportunities [14].

At present, in the pathogenesis of IBD, the role of dysregulation of the Th17/Treg axis has been particularly emphasized. Th17 and Treg cells reside mainly in the gut mucosa [1]. In the state of homeostasis, the Th17 cell lineage maintains the immune response necessary to protect the host against pathogens, whereas the Treg cell lineage controls Th17 cells and inhibits their excessive immune response. Thus, there is a delicate Th17/Treg balance. In turn, in the inflammatory milieu, excessive activation of Th17 cells and deficiency of Treg cells occur. Such a shift in the Th17/Treg equilibrium towards the proinflammatory Th17 side causes an uncontrolled immune response and massive intestinal inflammation [1,6].

Th17 and Treg cells develop from naïve CD4^+^ T cells depending on the presence of lineage-specific transcription factors and microenvironmental cues [6,15]. Th17 cell differentiation commences with IL-6 signaling, which is crucial to promote in naïve CD4^+^ T cells RORγt and STAT3 expression [16,17]. Additionally, in newly differentiating Th17 cells, IL-6, together with RORγt, promote IL-23 receptor (IL-23R) expression, rendering them receptive to IL-23 signaling. Although IL-23 is not needed for Th17 differentiation, it is essential for their pathogenicity [6,16]. Lamina propria CD4^+^ T cells from the intestines of mice with a lack of IL-6 show faulty expression of RORγt, IL-17, and IL-23R [18]. Upon stimulation with IL-6, STAT3, one of the STAT family of transcription factors members present in the cytoplasm in an inactive form, is activated by phosphorylation at tyrosine residue 705 (Y705). Phosphorylated STAT3 dimerizes, translocates to the nucleus, and regulates the expression of genes involved in the development and function of Th17, such as *Rorc*, *Il17*, and *Il23r* [19]. Many studies have shown that overexpression of STAT3 promotes the development of the Th17 lineage, and this process is strongly impaired in murine STAT3-deficient T cells [15,20]. In turn, IL-6-driven STAT3 signaling is required for the expression of a specific transcript of the *Rorc* gene, namely RORγt. Finally, RORγt and STAT3 bind to the promoter region of the *Il17* gene and lead to the expression of IL-17, the signature cytokine of Th17 cells. RORγt-deficient mice show impaired Th17 differentiation and IL-17 production [20]. The impaired ROR𝛾t expression leads to the increased level of Foxp3, a transcription factor required for the polarization and function of the Treg lineage [1,6]. Noteworthy, Foxp3 is able to inhibit the differentiation and function of the Th17 lineage by directly antagonizing the function of RORγt [21]. Contrary, IL-6 overcomes this suppressive effect of Foxp3 and induces a redifferentiation program in Foxp3^+^ Treg cells. IL-6 can reprogram fully differentiated Treg cells and re-differentiate them toward the Th17 lineage, a process also mediated by STAT3 [22].

Given the evidence for the importance of IL-6, RORγt, total STAT3 and its activated form *p*-STAT3, and Foxp3 levels in the immunoregulation of the Th17/Treg axis and their correlation with the severity of both experimental colitis and human IBD [6,23,24,25,26], it can be assumed that these factors may be potential targets in the pharmacotherapy of IBD and normalization of their levels may lead to prevention or at least alleviation the symptoms of intestinal inflammation. This kind of effect has been revealed in the current study, and to the best of our knowledge, this is the first study showing that cornelian cherry extract may influence specific molecules of the Th17/Treg axis developmental pathway. We have shown herein that pretreatment with CE at a dose of 100 mg/kg, but not at a dose of 20 mg/kg, normalized TNBS-induced increase of IL-6 and RORγt concentration, suppressed phosphorylation of STAT3 at Tyr705, and concomitantly counteracted the decrease in Foxp3 concentration in the colon tissues. This action may be the mechanism underlying the beneficial effect of the studied extract in TNBS-induced colitis demonstrated in our previous study, in which cornelian cherry extract alleviated the symptoms of experimental colitis, resulting in significant improvements at morphological, histological, and biochemical levels in the colon tissues [12]. The results achieved in this study suggest that cornelian cherry extract exerted a beneficial anti-inflammatory effect both by inhibiting the IL-6/STAT3/RORγt and promoting the Foxp3 signaling pathways, which means that an important property of cornelian cherry extract is its multidirectional action. Targeting the Th17-specific regulatory proteins—controlling both Th17 differentiation and Th17-related effector cytokines gene transcription—could, concomitantly, inhibit the production of several Th17-related effector cytokines acting synergistically to induce tissue inflammation [27]. This may explain our earlier findings showing that CE decreased Th17-related effector cytokines levels, i.e., IL-17, TNF-α, and chemerin [12]. Apart from cytokines, IL-23R is also under transcriptional control of RORγt [6]. In IBD, IL-23/IL-23R signaling is required for Th17 cells to reveal their pathogenic proinflammatory nature [16]. Thus, it may imply that CE decreased the production of gut-pathogenic IL-23-dependent IL-17 via downregulating the RORγt expression. In turn, the up-regulated Foxp3 can reverse Th17 cell development by binding and inactivating RORγt, thus favoring Treg development. Since the presence of proinflammatory molecules, especially IL-6, can revoke this Foxp3-mediated inhibition of RORγt via phosphorylation of STAT3 at Tyr705 [22], a concomitant increase in Foxp3 and decrease in IL-6 and RORγt concentration, and decrease the activation of STAT3 exerted by CE suggest that this investigated extract can not only prevent excessive differentiation of Th17 but also re-differentiation of Treg into pathogenic Th17 cells.

It is noteworthy that the Th17/Treg cell developmental pathway also has some additional modulators, e.g., the peroxisome proliferator-activated receptor γ (PPARγ) [28] or PIAS3 [29]. PPARγ negatively regulates Th17 cell differentiation through downregulation of the IL-6/STAT3/RORγt signaling pathway [28]. As earlier demonstrated, cornelian cherry iridoid-polyphenolic extract increased the expression of the PPARγ receptor [30,31]. It may imply that the therapeutic effect of CE against colitis and its action on the Th17/Treg developmental pathway is due to the activation of the PPARγ receptor. Another factor that negatively regulates Th17 cell differentiation and thereby exerts inhibitory effects on the progression of gut inflammation is PIAS3 [29]. PIAS3 is able to specifically interact with the phosphorylated STAT3 and inhibit its transcriptional activity by blocking the DNA-binding activity of *p*-STAT3 with its target genes. Physiologically, activation of STAT3 is rapid and transient since it is negatively regulated by PIAS3. In turn, in the intestinal tissues of IBD patients, STAT3 is persistently activated. Thus, PIAS3 up-regulation may lead to the abrogation of the overactivation of STAT3 in the disease state [8]. In the current study, we have shown that contrary to sulfasalazine, cornelian cherry extract at a higher dose elevated PIAS3 mRNA expression. However, it was not reflected in the greater action of the studied extract than sulfasalazine on STAT3 phosphorylation. Interestingly, Dalmasso et al. [32] demonstrated that adherent-invasive *E. coli* (AIEC) strain LF82, which is associated with CD, decreased PIAS3 level. In our previous paper, we have shown bacteriostatic and antiadhesive activities of CE against AIEC LF82 strains [12]. This antimicrobial activity of CE might explain, at least in part, an increase in PIAS3 mRNA expression in CE-pretreated rats.

The results of the present study are in line with many other studies demonstrating that among compounds that influence Th17/Treg axis balance via targeting its developmental or regulatory molecules are various phytocompounds, including polyphenols [9]. Some compounds present in the phenolic part of the studied extract have been proven to influence the Th17/Treg axis. Extract of *Terminalia catappa* containing phenolic acids, especially ellagic acid, a compound present also in CE, reduced the colonic expression of IL-6 in murine models of colitis [33]. Similarly, *Aronia* berry extract, rich in phenolic acids and anthocyanins, lowered IL-6 levels in DSS-induced colitis in mice [34]. *Pistacia lentiscus* extract, containing an abundance of phenolic compounds, including those found in CE, namely caffeoylquinic acid and kaempferol glucosides, also decreased the level of IL-6 in experimental colitis [35]. As demonstrated by Marin et al. and Saadatdoust et al. [36,37], a single compound, ellagic acid, and cocoa-derived phenolic compounds decreased colonic IL-6 levels and then STAT3 activation, as reflected in *p*-STAT3-Y705 expression in the DSS model of mice colitis. In turn, both the single anthocyanin compounds (cyanidin, cyanidin glycosides, pelargonidin) and anthocyanin-rich extract alleviated the clinical symptoms of colitis, decreasing the expression of IL-6 and increasing the expression of Foxp3 in the colon tissues, and promoting Treg cell expansion [38,39,40,41]. Moreover, cyanidin has been shown to activate PIAS3 protein to suppress STAT3-specific transcriptional activation [42]. Wei et al. [43] showed that quercetin, a flavonol-group phytocompound also present in cornelian cherry extract, suppressed RORγt and increased Foxp3 levels in liver injury associated with Th17/Treg imbalance in mice.

Phytocompounds are of value as adjuvants that may increase the effectiveness of standard pharmacotherapy in the case of IBD, e.g., with sulfasalazine. Such synergistic action was observed when the cornelian cherry extract was administered concomitantly with sulfasalazine. The combined therapy with CE at a lower dose and SA was more effective than SA alone, only in reversing the TNBS-induced decrease in Foxp3 level. It suggests the synergistic action of concomitantly administered CE at a lower dose and sulfasalazine only on the Treg cell development pathway. In turn, the combined therapy with cornelian cherry extract at a higher dose and sulfasalazine counteracted all analyzed TNBS-induced changes in a more effective way than sulfasalazine alone. It indicates the synergistic interaction between the higher dose CE combined with SA on both Th17 and Treg developmental pathways. The more pronounced effect shown by, the higher dose CE in combination with SA can be explained by the fact that, unlike SA, the higher dose CE combined with SA normalized the levels of RORγt, total STAT3, and PIAS3 mRNA. Such a strong effect of this combined therapy may be due to the increase in PIAS3 expression since only the higher dose CE alone or combined with SA caused the elevation in PIAS3 expression. Moreover, it is worth emphasizing that only combined therapy with cornelian cherry extract at a higher dose and sulfasalazine affected total STAT3 concentration and *p*-STAT3/STAT3 ratio. The achieved results concur well with our earlier findings that cornelian cherry extract administered concomitantly with sulfasalazine counteracted colitis more effectively than sulfasalazine alone, accompanied by a more effective reversion of TNBS-elevated concentrations of Th17-related cytokines such as IL-17 and TNF-α [12]. The current study is the first to assess the effect of concomitantly administered cornelian cherry iridoid-polyphenolic extract and sulfasalazine at a dose corresponding to that used in humans, demonstrating that such combined therapy counteracted the TNBS-induced changes in the levels of specific molecules of the Th17/Treg developmental pathway.

## 4. Materials and Methods

The current study was carried out on the biobank colon tissue samples collected during the previous original experiment [12]. The design of the original study and the methods used in the current study are described below.

### 4.1. In Vivo Experiment, Colon Tissue Samples Collection

The current study was carried out on the biobank colon tissue samples collected during the previous original experiment approved by the Local Ethics Committee for Experiments on Animals in Wrocław (No. 17/2017) and described in detail in the earlier paper [12]. Shortly, the experiment was performed on male Wistar rats weighing 220–260 g, housed two in a cage under standard and controlled laboratory conditions (light cycle, temperature, humidity, enrichments, free access to water and food, except for one procedure of deprivation). In the original study, rats were divided into 11 groups of 7–8 animals; however, in the current research, the colon tissue samples collected from groups receiving loganic acid at doses of 10 or 50 mg/kg with or without reference sulfasalazine (100 mg/kg) were not assessed. This was based on earlier observations that loganic acid did not exert significant protection in experimental colitis [12].

The material was analyzed from the following experimental groups: the control group receiving distilled water intragastrically (*i.g.*) and once saline per rectum (*p.r.*), the colitis (TNBS) group receiving distilled water *i.g.* and once 2,4,6-trinitrobenzenesulfonic acid (TNBS) solution *p.r.*, and 5 groups receiving cornelian cherry extract (at doses of 20 or 100 mg/kg) or sulfasalazine (100 mg/kg) or cornelian cherry extract (at doses of 20 or 100 mg/kg) with sulfasalazine (100 mg/kg) and once TNBS solution *p.r.* (CE20, CE100, SA, CE20+SA, CE100+SA groups, respectively).

Distilled water, CE, or SA, was given once daily by a gastric tube (FST, Foster City, CA, USA) for 16 days. Normal saline or TNBS solution (Sigma-Aldrich, Steinheim, Germany) was given rectally on the 15th day of the experiment, after 24 h of food deprivation. The induction of colitis was performed following the procedure first described by Morris et al. [44], detailed in our previous paper [12]. It involved anesthesia with ketamine (75 mg/kg) and medetomidine (0.5 mg/kg), placement of the animal on its right side, application into the colon (8 cm from the anus), the ethanol solution (50%, *v/v*) of TNBS (50 mg/kg) by the polyethylene tube (Ø 2 mm) and keeping the animal for 5 min in Trendelenburg position to prevent leakage of the applied solution. Rats were euthanized by cervical dislocation under pentobarbital (200 mg/kg) anesthesia 48 h after colitis induction. After completing the experiment, the distal, 8 cm long sections of the colon were resected, cleaned, weighed, portioned, and properly prepared for future analyses.

### 4.2. Plant Material and Extraction

The cornelian cherry fruits of the ‘Raciborski’ cultivar were collected from the Arboretum and Institute of Physiography in Bolestraszyce (49°49′01.1″ N, 22°51′24.2″ E). The plant material was authenticated by Prof. Narcyz Piórecki. The voucher specimens (BDPA 3967) have been deposited at the Herbariums of Arboretum in Bolestraszyce, Poland. After harvesting, fully ripe red fruits were immediately frozen at −20 °C until further analysis. After defrosting the fruit, the juice was obtained, according to Kucharska et al. [45]. The pressed juice was purified on an Amberlite XAD-16 resin column (Rohm and Haas, Chauny Cedex, France). Impurities were washed off with distilled water. Extract from red juice was eluted with 80% ethanol (POCh, Gliwice, Poland). The cornelian cherry extract was concentrated under a vacuum at 40 °C. Rotavapor (Unipan, Warszawa, Poland) was used to evaporate the solvent. Next, the cornelian cherry extract was freeze-dried (Alpha 1–4 LSC, Christ, Osterode am Harz, Germany).

### 4.3. Chemical Analysis of Cornelian Cherry Iridoid-Polyphenolic Extract

The chemical analysis of the cornelian cherry extract was performed by high-performance liquid chromatography (HPLC-PDA), as described previously by Kucharska et al. [46]. The HPLC analysis was performed using a Dionex (Germering, Germany) system equipped with an Ultimate 3000 diode array detector, LPG-3400A quaternary pump, EWPS-3000SI autosampler, TCC-3000SD thermostated column compartment, and controlled by Chromeleon v.7.2 software (Thermo Fisher, Waltham, MA, USA). The HPLC conditions are summarized in Table S1. The compounds were identified by comparing their retention times and UV-Vis spectra with authentic standards of iridoid and phenolic compounds (Extrasynthese, Genay, France) and by comparison with literature data. Iridoids were quantified as loganic acid, anthocyanins as cyanidin 3-*O*-glucoside, phenolic acids as *p*-coumaric acid, caffeic acid, and ellagic acid, flavonols as quercetin 3-*O*-glucoside or kaempferol 3-*O*-glucoside. The cornelian cherry extract was analyzed in three repetitions.

### 4.4. Preparation of Colon Tissue Homogenates

The colon tissues were rinsed in 1 × PBS to remove excess blood, cut into small pieces, and homogenized (Homogenizer Pro250 Pro Scientific Inc., Oxford, CT, USA) on ice in 1 × PBS. After two freeze-thaw cycles, the homogenates were centrifuged (4000 rpm, 10 min, 4 °C) using an MPW-350R laboratory centrifuge (MPW Med. Instruments, Warszawa, Poland), and supernatants were collected, aliquoted, and stored at −80 °C until the analyses.

### 4.5. IL-6, RORγt, Total STAT3, Phosphorylated STAT3 (p-STAT3), and Foxp3 Quantification by Enzyme-Linked Immunosorbent Assay (ELISA) 

In colon tissue homogenates, IL-6, RORγt, and Foxp3 were analyzed using the Rat IL-6 ELISA Kit, Rat RORγt ELISA Kit, and Rat Foxp3 ELISA Kit, respectively (ELK Biotechnology CO., Ltd., Wuhan, China). Total STAT3 and phosphorylated STAT3 (*p*-STAT3) were assessed using Rat Phospho-Stat3 (Y705) and Pan Stat3 ELISA Kit (RayBiotech Inc., Norcross, GA, USA). All parameters were assayed following the manufacturers’ instructions. The concentration of IL-6 was given in pg/mL, and the levels of RORγt, total STAT3, *p*-STAT3, and Foxp3 were expressed in ng/mL. Total STAT3 and *p*-STAT3 levels were also expressed as the *p*-STAT3/STAT3 ratio.

### 4.6. RNA Isolation, Quantification and Reverse Transcription

Until RNA isolation, colon tissue samples were soaked in RNAlater (Ambion Inc., Carlsbad, CA, USA) and kept at −80 °C. Tissue specimens (up to 40 mg) were homogenized (Fastprep 24 Homogenizer, MP Biomedical, Solon, OH, USA) in lysis buffer (Thermo-Fisher Scientific, Waltham, MA, USA) supplemented with β-mercaptoethanol (Sigma-Aldrich, Saint Louis, MO, USA). RNA was isolated by phenol-chloroform extraction followed by purification with PureLink™ RNA Mini Kit (Thermo-Fisher Scientific, Waltham, MA, USA). An on-column treatment of RNA isolates with DNase (PureLink™ DNase Set, Invitrogen™, Thermo-Fisher Scientific, Waltham, MA, USA) was used to remove genomic DNA. Isolated and purified RNA was quantified using NanoDrop 2000 (Thermo-Fisher Scientific, Waltham, MA, USA), and purity was evaluated by calculating ratios of absorbance at 260, 280, and 230 nm. Then, 1 µg of RNA per 20 µL reaction mixture was reversely transcribed using a C1000 thermocycler (BioRad, Hercules, CA, USA) and the iScript™ cDNA Synthesis Kit (BioRad, Hercules, CA, USA) according to the manufacturer’s instructions. All studied samples were accompanied by matching “no-RT” (negative transcription) controls lacking reverse transcriptase, then tested to ensure that there was no genomic DNA contamination.

### 4.7. Quantitative Real-Time Polymerase Chain Reaction (qPCR)

CFX96 Real-Time PCR system (BioRad, Hercules, CA, USA) and SsoFast EvaGreen^®^ Supermix (BioRad, Hercules, CA, USA) were used to carry out quantitative (real-time) PCR (qPCR) reactions. The cycling conditions were as follows: 30-sec activation (95 °C), 5-sec denaturation (95 °C), 5-sec annealing/extension (61 °C), 40 cycles, followed by a melting step (60–95 °C with fluorescent reading every 0.5 °C). The reaction mixture was composed of 2 µL of diluted 1:7.5 cDNA, 10 µL of 2× SsoFast EvaGreen^®^ Supermix, 1 µL of each 10 nM forward and reverse target-specific primers, and water up to 20 µL. Gene name, accession number, and the sequence of studied primers are shown in Table 2. Primers were synthesized by Genomed (Warszawa, Poland) and their specificities were tested by melting curve analysis and electrophoresis in high-resolution SeaKem LE agarose (Lonza, Basel, Switzerland) in TBE with SYBR Green (Lonza, Basel, Switzerland) detection. Technical replicates were averaged before expression analysis. A normalized relative quantity (NRQ) of a given gene transcript for each sample was calculated with qbasePLUS software version 2.4 (Biogazelle BE, Ghent, Belgium). The NRQ was calculated as a 2^(ΔCq: the difference between the geometric mean of Cq in the whole group—sample Cq) normalized to GAPDH, serving as an internal control.

### 4.8. Statistical Analysis

After confirming the normality of data distribution and equality of variance by the Shapiro-Wilk and Brown-Forsythe tests, respectively, the analysis of the obtained results was performed by the one-way ANOVA, followed by Tukey’s *post hoc* test (The GraphPad Prism version 8.0 was used (GraphPad Software, San Diego, CA, USA). The level of significance to verify the zero hypothesis was assessed as *p* < 0.05.

## 5. Conclusions

The cornelian cherry extract regulates the Th17/Treg developmental pathway molecules, and this effect may be related to its polyphenolic compounds. This action may underlie a protective effect of the studied cornelian cherry extract in experimental colitis. The combined pretreatment with cornelian cherry extract and sulfasalazine is more effective than sulfasalazine alone. This suggests a synergistic action of CE and SA on the crucial developmental factors of the Th17/Treg axis and confirms the possibility of using the studied cornelian cherry extract in adjuvant therapy in IBD.

## Figures and Tables

**Table 1:** The impact of cornelian cherry iridoid-polyphenolic extract on total STAT3 and activated (phosphorylated at Tyr705) STAT3 (*p*-STAT3) levels in the colon tissues in experimental groups.

Parameter	Group	Value	*p*-Value (vs. Control)	*p*-Value (vs. TNBS)	*p*-Value (vs. SA)	*p*-Value (vs. CE100)
Total STAT3 (ng/mL)	Control	43.61 ± 10.01	-	0.0015	NS	NS
	TNBS	64.05 ± 10.48	0.0015	-	NS	NS
	CE20	61.39 ± 13.01	0.0114	NS	NS	NS
	CE100	49.71 ± 8.08	NS	NS	NS	-
	SA	55.47 ± 9.82	NS	NS	-	NS
	CE20 + SA	59.03 ± 8.38	0.0414	NS	NS	NS
	CE100 + SA	39.32 ± 4.71	NS	<0.0001	0.0283	NS
*p*-STAT3 (ng/mL)	Control	9.32 ± 1.75	-	<0.0001	<0.0001	0.0033
	TNBS	23.06 ± 5.50	<0.0001	-	0.0167	<0.0001
	CE20	19.30 ± 2.23	<0.0001	NS	NS	NS
	CE100	15.04 ± 2.81	0.0033	<0.0001	NS	-
	SA	18.06 ± 2.61	<0.0001	0.0167	-	NS
	CE20 + SA	17.49 ± 1.51	<0.0001	0.0061	NS	NS
	CE100 + SA	8.96 ± 1.69	NS	<0.0001	<0.0001	0.0017
*p*-STAT3/total STAT3 ratio	Control	0.22 ± 0.07	-	0.0113	NS	NS
	TNBS	0.36 ± 0.08	0.0113	-	NS	NS
	CE20	0.33 ± 0.11	NS	NS	NS	NS
	CE100	0.31 ± 0.05	NS	NS	NS	-
	SA	0.34 ± 0.10	NS	NS	-	NS
	CE20 + SA	0.30 ± 0.05	NS	NS	NS	NS
	CE100 + SA	0.23 ± 0.06	NS	0.0201	NS	NS

Notes: Control—control group; TNBS—group receiving only TNBS; CE20, CE100—groups receiving, respectively, 20 or 100 mg/kg cornelian cherry extract and TNBS; SA—group receiving 100 mg/kg sulfasalazine and TNBS; CE20 + SA, CE100 + SA—groups receiving, respectively, 20 or 100 mg/kg cornelian cherry extract and 100 mg/kg sulfasalazine and TNBS. Data are presented as mean values ± SD. Analyses were performed using a one-way ANOVA and Tukey’s *post hoc* test; *p*-value < 0.05 was deemed as the significance level; NS, not significant.

**Table 2:** Sequences of primers used in the current study.

Symbol	Gene Name	Accession No.	Primer Sequence 5′→ 3′	Amp. Size
*Gapdh*	Glyceraldehyde-3-phosphate dehydrogenase	NM_017008.4	F: tgactctacccacggcaagttcaa R: acgacatactcagcaccagcatca	141 bp
*Pias3*	Protein inhibitor of activated STAT3	NM_031784.2	F: ttcgctggcaggaacaagag R: gggcgcagctagacttgag	79 bp

Notes: The sequences of studied primers were retrieved from scientific literature and validated in silico by Blast analysis. The forward and reverse primer sequences are labeled “F” and “R”, respectively. Amp., amplicon; bp, base pairs.

## Data Availability

The data underlying this article will be shared upon request to the corresponding author.

## Supplementary Materials

The following supporting information can be downloaded at: https://www.mdpi.com/article/10.3390/molecules28073034/s1, Table S1: The HPLC conditions used for iridoid and phenolic compounds analysis. Figure S1: HPLC-PDA chromatograms of compounds of the purified cornelian cherry iridoid-polyphenolic extract (CE) at 245 nm (two iridoids: (1) LA–loganic acid, (2) Co–cornuside), 520 nm (seven anthocyanins: (1) Df gal–delphinidin 3-O-galactoside; (2) Cy gal–cyanidin 3-O-galactoside; (3) Cy rob–cyanidin 3-O-robinobioside; (4) Pg gal–pelargonidin 3-O-galactoside; (5) Pg rob–pelargonidin 3-O-robinobioside; (6) Cy–cyanidin; (7) Pg–pelargonidin), 360 nm (four flavonols: (1) Q glrd–quercetin 3-O-glucuronide; (2) Q glc–quercetin 3-O-glucoside; (3) Kf glrd–kaempferol 3-O-glucuronide; (4) Kf gal–kaempferol 3-O-galactoside), and 320 nm (seven phenolic acids: (1) Cahex–caffeoylhexoside; (2) *p*-CuQA 1–*p*-coumaroilquinic acid 1; (3) CaQA– caffeoylquinic acid; (4) *p*-CuA–*p*-coumaric acid; (5) *p*-CuQA 2–*p*-coumaroilquinic acid 2; (6) *p*-CuQA 3–*p*-coumaroilquinic acid 3; (7) EA–ellagic acid).

## Sample Availability

Samples of the studied cornelian cherry extract are available from the authors.

## References

[1] Gálvez J. (2014). Role of Th17 Cells in the Pathogenesis of Human IBD. ISRN Inflamm..

[2] Ananthakrishnan A.N., Kaplan G.G., Ng S.C. (2020). Changing Global Epidemiology of Inflammatory Bowel Diseases: Sustaining Health Care Delivery into the 21st Century. Clin. Gastroenterol. Hepatol..

[3] Lönnfors S., Vermeire S., Greco M., Hommes D., Bell C., Avedano L. (2014). IBD and Health-Related Quality of Life—Discovering the True Impact. J. Crohns Colitis.

[4] Seyedian S.S., Nokhostin F., Malamir M.D. (2019). A Review of the Diagnosis, Prevention, and Treatment Methods of Inflammatory Bowel Disease. J. Med. Life.

[5] Szandruk-Bender M., Merwid-Ląd A., Wiatrak B., Danielewski M., Dzimira S., Szkudlarek D., Szczukowski Ł., Świątek P., Szeląg A. (2021). Novel 1,3,4-Oxadiazole Derivatives of Pyrrolo[3,4-d]Pyridazinone Exert Anti-Inflammatory Activity without Acute Gastrotoxicity in the Carrageenan-Induced Rat Paw Edema Test. J. Inflamm. Res..

[6] Yan J.-B., Luo M.-M., Chen Z.-Y., He B.-H. (2020). The Function and Role of the Th17/Treg Cell Balance in Inflammatory Bowel Disease. J. Immunol. Res..

[7] Noack M., Miossec P. (2014). Th17 and Regulatory T Cell Balance in Autoimmune and Inflammatory Diseases. Autoimmun. Rev..

[8] Yagil Z., Nechushtan H., Kay G., Yang C.M., Kemeny D.M., Razin E. (2010). The Enigma of the Role of Protein Inhibitor of Activated STAT3 (PIAS3) in the Immune Response. Trends Immunol..

[9] Chang Y., Zhai L., Peng J., Wu H., Bian Z., Xiao H. (2021). Phytochemicals as Regulators of Th17/Treg Balance in Inflammatory Bowel Diseases. Biomed. Pharm..

[10] Fasching P., Stradner M., Graninger W., Dejaco C., Fessler J. (2017). Therapeutic Potential of Targeting the Th17/Treg Axis in Autoimmune Disorders. Molecules.

[11] Szandruk-Bender M., Wiatrak B., Dzimira S., Merwid-Ląd A., Szczukowski Ł., Świątek P., Szeląg A. (2022). Targeting Lineage-Specific Transcription Factors and Cytokines of the Th17/Treg Axis by Novel 1,3,4-Oxadiazole Derivatives of Pyrrolo[3,4-d]Pyridazinone Attenuates TNBS-Induced Experi-Mental Colitis. Int. J. Mol. Sci..

[12] Szandruk-Bender M., Rutkowska M., Merwid-Ląd A., Wiatrak B., Szeląg A., Dzimira S., Sobieszczańska B., Krzystek-Korpacka M., Kucharska A.Z., Matuszewska A. (2020). Cornelian Cherry Iridoid-Polyphenolic Extract Improves Mucosal Epithelial Barrier Integrity in Rat Experimental Colitis and Exerts Antimicrobial and Antiadhesive Activities In Vitro. Oxid. Med. Cell. Longev..

[13] Dziąbowska-Grabias K., Sztanke M., Zając P., Celejewski M., Kurek K., Szkutnicki S., Korga P., Bulikowski W., Sztanke K. (2021). Antioxidant Therapy in Inflammatory Bowel Diseases. Antioxidants.

[14] Boyapati R.K., Kalla R., Satsangi J., Ho G.-T. (2016). Biomarkers in Search of Precision Medicine in IBD. Am. J. Gastroenterol..

[15] Garrido-Mesa N., Algieri F., Rodríguez Nogales A., Gálvez J. (2013). Functional Plasticity of Th17 Cells: Implications in Gastrointestinal Tract Function. Int. Rev. Immunol..

[16] Patel D.D., Kuchroo V.K. (2015). Th17 Cell Pathway in Human Immunity: Lessons from Genetics and Therapeutic Interventions. Immunity.

[17] Zhao J., Lu Q., Liu Y., Shi Z., Hu L., Zeng Z., Tu Y., Xiao Z., Xu Q. (2021). Th17 Cells in Inflammatory Bowel Disease: Cytokines, Plasticity, and Therapies. J. Immunol. Res..

[18] Zhou L., Ivanov I.I., Spolski R., Min R., Shenderov K., Egawa T., Levy D.E., Leonard W.J., Littman D.R. (2007). IL-6 Programs T(H)-17 Cell Differentiation by Promoting Sequential Engagement of the IL-21 and IL-23 Pathways. Nat. Immunol..

[19] Rébé C., Végran F., Berger H., Ghiringhelli F. (2013). STAT3 Activation. JAKSTAT.

[20] Korn T., Bettelli E., Oukka M., Kuchroo V.K. (2009). IL-17 and Th17 Cells. Annu. Rev. Immunol..

[21] Ueno A., Ghosh A., Hung D., Li J., Jijon H. (2015). Th17 Plasticity and Its Changes Associated with Inflammatory Bowel Disease. World J. Gastroenterol..

[22] Yang X.O., Nurieva R., Martinez G.J., Kang H.S., Chung Y., Pappu B.P., Shah B., Chang S.H., Schluns K.S., Watowich S.S. (2008). Molecular Antagonism and Plasticity of Regulatory and Inflammatory T Cell Programs. Immunity.

[23] Abraham C., Dulai P.S., Vermeire S., Sandborn W.J. (2017). Lessons Learned From Trials Targeting Cytokine Pathways in Patients With Inflammatory Bowel Diseases. Gastroenterology.

[24] Buchele V., Konein P., Vogler T., Kunert T., Enderle K., Khan H., Büttner-Herold M., Lehmann C.H.K., Amon L., Wirtz S. (2020). Th17 Cell-Mediated Colitis Is Positively Regulated by Interferon Regulatory Factor 4 in a T Cell-Extrinsic Manner. Front. Immunol..

[25] Neurath M.F. (2014). Cytokines in Inflammatory Bowel Disease. Nat. Rev. Immunol..

[26] Yamada A., Arakaki R., Saito M., Tsunematsu T., Kudo Y., Ishimaru N. (2016). Role of Regulatory T Cell in the Pathogenesis of Inflammatory Bowel Disease. World J. Gastroenterol..

[27] Yang J., Sundrud M.S., Skepner J., Yamagata T. (2014). Targeting Th17 Cells in Autoimmune Diseases. Trends Pharmacol. Sci..

[28] Park B.V., Pan F. (2015). The Role of Nuclear Receptors in Regulation of Th17/Treg Biology and Its Implications for Diseases. Cell Mol. Immunol..

[29] Min H.-K., Choi J., Lee S.-Y., Seo H.-B., Jung K., Na H.S., Ryu J.-G., Kwok S.-K., Cho M.-L., Park S.-H. (2019). Protein Inhibitor of Activated STAT3 Reduces Peripheral Arthritis and Gut Inflammation and Regulates the Th17/Treg Cell Imbalance via STAT3 Signaling in a Mouse Model of Spondyloarthritis. J. Transl. Med..

[30] Sozański T., Kucharska A., Dzimira S., Magdalan J., Szumny D., Matuszewska A., Nowak B., Piórecki N., Szeląg A., Trocha M. (2018). Loganic Acid and Anthocyanins from Cornelian Cherry (*Cornus mas* L.)Fruits Modulate Diet-Induced Atherosclerosis and Redox Status in Rabbits. Adv. Clin. Exp. Med..

[31] Danielewski M., Matuszewska A., Nowak B., Kucharska A.Z., Sozański T. (2020). The Effects of Natural Iridoids and Anthocyanins on Selected Parameters of Liver and Cardiovascular System Functions. Oxidative Med. Cell. Longev..

[32] Dalmasso G., Nguyen H.T.T., Faïs T., Massier S., Barnich N., Delmas J., Bonnet R. (2019). Crohn’s Disease-Associated Adherent-Invasive Escherichia Coli Manipulate Host Autophagy by Impairing SUMOylation. Cells.

[33] Abiodun O.O., Rodríguez-Nogales A., Algieri F., Gomez-Caravaca A.M., Segura-Carretero A., Utrilla M.P., Rodriguez-Cabezas M.E., Galvez J. (2016). Antiinflammatory and Immunomodulatory Activity of an Ethanolic Extract from the Stem Bark of *Terminalia catappa* L. (*Combretaceae*): In Vitro and in Vivo Evidences. J. Ethnopharmacol..

[34] Kang S.-H., Jeon Y.-D., Moon K.-H., Lee J.-H., Kim D.-G., Kim W., Myung H., Kim J.-S., Kim H.-J., Bang K.-S. (2017). Aronia Berry Extract Ameliorates the Severity of Dextran Sodium Sulfate-Induced Ulcerative Colitis in Mice. J. Med. Food.

[35] Zahouani Y., Ben Rhouma K., Kacem K., Sebai H., Sakly M. (2021). Aqueous Leaf Extract of Pistacia Lentiscus Improves Acute Acetic Acid-Induced Colitis in Rats by Reducing Inflammation and Oxidative Stress. J. Med. Food.

[36] Marín M., María Giner R., Ríos J.-L., Carmen Recio M. (2013). Intestinal Anti-Inflammatory Activity of Ellagic Acid in the Acute and Chronic Dextrane Sulfate Sodium Models of Mice Colitis. J. Ethnopharmacol..

[37] Saadatdoust Z., Pandurangan A.K., Ananda Sadagopan S.K., Mohd Esa N., Ismail A., Mustafa M.R. (2015). Dietary Cocoa Inhibits Colitis Associated Cancer: A Crucial Involvement of the IL-6/STAT3 Pathway. J. Nutr. Biochem..

[38] Biagioli M., Carino A., Fiorucci C., Annunziato G., Marchianò S., Bordoni M., Roselli R., Giorgio C.D., Castiglione F., Ricci P. (2019). The Aryl Hydrocarbon Receptor (AhR) Mediates the Counter-Regulatory Effects of Pelargonidins in Models of Inflammation and Metabolic Dysfunctions. Nutrients.

[39] Gan Y., Fu Y., Yang L., Chen J., Lei H., Liu Q. (2020). Cyanidin-3-O-Glucoside and Cyanidin Protect Against Intestinal Barrier Damage and 2,4,6-Trinitrobenzenesulfonic Acid-Induced Colitis. J. Med. Food.

[40] Kim Y.-J., Ju J., Song J.-L., Yang S., Park K.-Y. (2018). Anti-Colitic Effect of Purple Carrot on Dextran Sulfate Sodium (DSS)-Induced Colitis in C57BL/6J Mice. Prev. Nutr. Food Sci..

[41] Xia Y., Tian L.-M., Liu Y., Guo K.-S., Lv M., Li Q.-T., Hao S.-Y., Ma C.-H., Chen Y.-X., Tanaka M. (2019). Low Dose of Cyanidin-3-O-Glucoside Alleviated Dextran Sulfate Sodium-Induced Colitis, Mediated by CD169+ Macrophage Pathway. Inflamm. Bowel Dis..

[42] Samarpita S., Ganesan R., Rasool M. (2020). Cyanidin Prevents the Hyperproliferative Potential of Fibroblast-like Synoviocytes and Disease Progression via Targeting IL-17A Cytokine Signalling in Rheumatoid Arthritis. Toxicol. Appl. Pharm..

[43] Wei C.-B., Tao K., Jiang R., Zhou L.-D., Zhang Q.-H., Yuan C.-S. (2017). Quercetin Protects Mouse Liver against Triptolide-Induced Hepatic Injury by Restoring Th17/Treg Balance through Tim-3 and TLR4-MyD88-NF-ΚB Pathway. Int. Immunopharmacol..

[44] Morris G.P., Beck P.L., Herridge M.S., Depew W.T., Szewczuk M.R., Wallace J.L. (1989). Hapten-Induced Model of Chronic Inflammation and Ulceration in the Rat Colon. Gastroenterology.

[45] Kucharska A.Z., Szumny A., Sokół-Łętowska A., Piórecki N., Klymenko S.V. (2015). Iridoids and Anthocyanins in Cornelian Cherry (*Cornus mas* L.) Cultivars. J. Food Compos. Anal..

[46] Kucharska A.Z., Sokół-Łętowska A., Oszmiański J., Piórecki N., Fecka I. (2017). Iridoids, Phenolic Compounds and Antioxidant Activity of Edible Honeysuckle Berries (*Lonicera caerulea var. kamtschatica sevast*.). Molecules.

