# Supplementary material for: Cornus mas L. Extract Targets the Specific Molecules of the Th17/Treg Developmental Pathway in TNBS-Induced Experimental Colitis in Rats
Source: Molecules. 2023 Mar 29;28(7):3034. doi: 10.3390/molecules28073034 (PMC10095994; doi:10.3390/molecules28073034)
Supplement: Supplementary file 1 [file molecules-28-03034-s001.zip › molecules-2249716-supplementary.pdf]

Supplementary Materials

Table S1. The HPLC conditions used for iridoid and phenolic compounds analysis.

	HPLC
Column	Cadenza column CD-C18 (75 × 4.6 mm, 5 μm)
Colum oven temprature	30 °C
Monitored wavelengths (nm)	245 (iridoids, ellagic acid), 320 (phenolic acid), 360 (flavonols), 520 (anthocyanins)
Mobile phases	A: 4.5% aq. formic acid, v/v<br>B: 100% acetonitrile
Gradient conditions	0 – 1 min 5% B in A, 1 – 20 min 25% B in A, 20 – 21 min 100% B, 21 – 26 min 100% B, 26 v 27 min 5% B in A
Flow rate (mL/min)	1.00
Injection volume (μL)	20

Notes: Acetonitrile and formic acid were obtained from POCh (Gliwice, Poland). Authentic standards of iridoid and phenolic compounds were purchased from Extrasynthese (Genay, France).

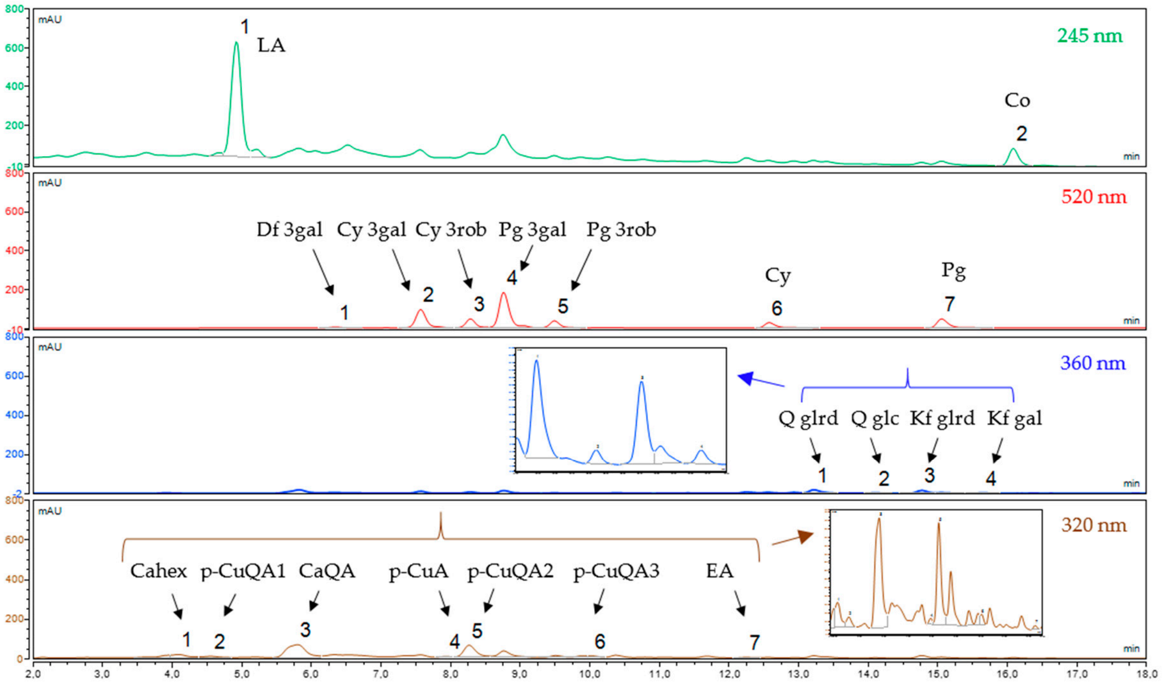

Figure S1. HPLC-PDA chromatograms of compounds of the purified cornelian cherry iridoid-polyphenolic extract (CE) at **245 nm** (two iridoids: **1) LA**–loganic acid, **2) Co**–cornuside), **520 nm** (seven anthocyanins: **1) Df gal**–delphinidin 3-*O*-galactoside; **2) Cy gal**–cyanidin 3-*O*-galactoside; **3) Cy rob**–cyanidin 3-*O*-robinobioside; **4) Pg gal**–pelargonidin 3-*O*-galactoside; **5) Pg rob**–pelargonidin 3-*O*-robinobioside; **6) Cy**–cyanidin; **7) Pg**–pelargonidin), **360 nm** (four flavonols: **1) Q glrd**–quercetin 3-*O*-glucuronide; **2) Q glc**–quercetin 3-*O*-glucoside; **3) Kf glrd**–kaempferol 3-*O*-glucuronide; **4) Kf gal**–kaempferol 3-*O*-galactoside), and **320 nm** (seven phenolic acids: **1) Cahex**–caffeoylhexoside; **2) *p*-CuQA 1**–*p*-coumaroilquinic acid 1; **3) CaQA**– caffeoylquinic acid; **4) *p*-CuA**–*p*-coumaric acid; **5) *p*-CuQA** 2–*p*-coumaroilquinic acid 2; **6) *p*-CuQA 3**–*p*-coumaroilquinic acid 3; **7) EA**–ellagic acid)
